# Donepezil combined with traditional Chinese medicine has promising efficacy on mild cognitive impairment: a systematic review and meta-analysis

**DOI:** 10.3389/fnins.2023.1206491

**Published:** 2023-07-05

**Authors:** Si-jia Yu, Hui-ling Tang, Wei-hong Li, Chen-li Bin, Zhang Liu, Zhao-hui Tang, Jing-hong Liang

**Affiliations:** ^1^Basic Medical College, Chengdu University of Traditional Chinese Medicine, Chengdu, China; ^2^Hospital of Chengdu University of Traditional Chinese Medicine, Chengdu, China; ^3^Department of Maternal and Child Health, School of Public Health, Sun Yat-sen University, Guangzhou, China

**Keywords:** mild cognitive impairment, traditional Chinese medicine, donepezil, meta-analysis, systematic review

## Abstract

**Objective:**

Prior research has shown mixed results regarding the effectiveness of combining donepezil and traditional Chinese medicine (TCM) to treat mild cognitive impairment (MCI). In light of this, our study aims to examine the efficacy and safety of this treatment approach for patients with MCI.

**Methods:**

We conducted a comprehensive search of various databases, including Medline (*via* PubMed), Cochrane, Embase, Web of Science, Chinese National Knowledge Infrastructure, Chinese Biomedical Literature Database, Chinese Scientific Journal Database, and Wanfang Database from their inception to November 16, 2022. The selection of studies, risk of bias assessment, and data extraction were carried out independently by two authors. The statistical analysis was performed using STATA.

**Results:**

Our meta-analysis included a total of 35 studies with 2,833 patients, published between 2008 and 2022, with intervention durations ranging from 4 weeks to 12 months. However, most of the studies had a high risk of detection bias. Our findings indicated that the combination of donepezil and TCM significantly improved the Montreal Cognitive Assessment (MoCA) score (weighted mean difference [WMD] = 2.79, 95% confidence interval [CI]: 1.82 to 3.75) and the Barthel Index score (WMD = 9.20, 95% CI: 5.39 to 13.00) compared to donepezil alone. However, subgroup analyses showed that the MoCA score did not increase significantly in patients with MCI resulting from cerebrovascular disease (WMD = 1.47, 95% CI: −0.02 to 2.96).

**Conclusion:**

The combination of donepezil and TCM may have a more positive effect on cognitive function and activities of daily living in patients with MCI compared to the use of donepezil alone. However, due to the limited quality of the studies included in our analysis, these findings should be interpreted with caution.

## 1. Introduction

Mild cognitive impairment (MCI) is a cognitive state that falls between normal aging and early dementia (Petersen, 2004). It is characterized by cognitive deficits in at least one domain accompanied by a slight decline in instrumental activities of daily living (Petersen et al., 2018). Along with this, MCI also causes decreased living quality, depression, and avoidant coping strategies like withdrawal from social engagement. These features are major indicators of MCI (Anderson, 2019). The prevalence of MCI tends to rise with age, ranging from 6.7% in individuals aged 60 to 65 to 25.2% in those aged 80 to 84 (Petersen et al., 2018). Furthermore, individuals with MCI have a higher risk of developing dementia compared to their age-matched healthy counterparts (Petersen, 2004; Petersen et al., 2018). A recent meta-analysis of epidemiological studies has revealed that the prevalence of MCI among the Chinese population aged 50 and above is 15.4% (Deng et al., 2021). With the global population aging rapidly, many countries, including China, are facing the challenge of MCI. By 2040, the number of senior citizens in China is projected to reach 402 million, accounting for approximately 28% of the population (Lancet, 2022). Therefore, it is crucial to prioritize interventions for MCI in order to improve the quality of life for patients, reduce the incidence of dementia, and alleviate the economic and medical burdens on society.

The demand for pharmacotherapy among patients with MCI and their families has increased significantly (Tricco et al., 2013). Donepezil, a commonly used Western medicine (WM) for improving cognition in MCI clinical research, has been found to have limited effects on cognitive function in patients with MCI and is associated with more adverse events (AEs), according to a recent meta-analysis (Zhang X. et al., 2022). Research on finding new treatments for patients with MCI has become a popular topic in recent times. In recent years, traditional Chinese medicine (TCM) has gained significant attention in treating MCI due to its better efficacy and fewer AEs (Pei et al., 2020). Modern studies have shown that TCM has multiple benefits in improving cognitive function, including regulating the central cholinergic system, reducing hippocampal oxidative stress, and protecting cerebral blood vessels (Pei et al., 2020). From an integrated WM and TCM perspective, we believe that the combination of donepezil and TCM may provide greater benefits in treating patients with MCI than donepezil alone. Research (Di et al., 2017) has shown that combining donepezil with *Ginkgo Biloba* extract tablets effectively improved cognitive function in patients with MCI and delayed the progression to Alzheimer’s disease (AD) when compared to donepezil alone. Similarly, another study (Shou et al., 2022) has found that combining donepezil with modified Shenghui decoction was superior to using donepezil alone in improving cognitive function and daily activities in patients with MCI. Several trials have suggested that the combination of donepezil and TCM may be more effective than donepezil alone in treating patients with MCI. However, the results of these trials are still controversial and inconclusive (Liu et al., 2008; Dong, 2015; Luo, 2019; Yang, 2021). Currently, there is no high-quality summary of the efficacy and safety of this combination therapy. This highlights the need for further research in this area. Previous meta-analyses investigating TCM on patients with MCI have had limitations that need to be addressed. First, previous studies on the efficacy of TCM on patients with MCI had methodological flaws that may have affected their results. For instance, some studies included participants with different conditions such as patients with MCI and dementia (Yang et al., 2016), or age-associated memory impairment (May et al., 2009). Additionally, other studies had diverse comparators including placebos, no intervention, and other therapies (Liang et al., 2022). These deficiencies were caused by the lack of strict eligibility criteria which resulted in high heterogeneity among the included studies and concealed the true efficacy of TCM on patients with MCI. Second, previous studies have mainly focused on the effects of TCM on cognitive function in patients with MCI, with limited attention given to other outcomes such as activities of daily living (Dong et al., 2019; Wang W. et al., 2021). This incomplete investigation of result indicators can hinder clinicians from fully understanding the effects of TCM on patients with MCI, thereby limiting their ability to make informed clinical decisions. Third, previous studies had limited published trials and participants (Wang W. et al., 2021) which may affect the reliability of their results. With the recent increase in relevant research (Li et al., 2022; Shou et al., 2022), it is necessary to update evidence. Therefore, we will use the meta-analysis method to quantify the efficacy and safety of donepezil combined with TCM on patients with MCI, guided by the theory of WM and TCM. This will provide more advanced, objective, and reliable evidence-based medical evidence for clinical decision-making and policy development. Due to the limitations of previous research on TCM in treating patients with MCI, we will conduct an extensive search, formulate rigorous inclusion criteria, and perform a more comprehensive and in-depth exploration.

## 2. Methods

Our study adhered to the 2020 Preferred Reporting Items for Systematic Reviews and Meta-Analyses (PRISMA; Page et al., 2021) and the Cochrane Handbook for Systematic Reviews of Interventions (Cumpston et al., 2019). As all data analyzed were from previous studies, ethical approval and patient consent were not required.

### 2.1. Literature search

To assess the effectiveness and safety of donepezil combined with TCM in treating patients with MCI, an extensive search was conducted in eight electronic databases: Medline (*via* PubMed), Cochrane, Embase, Web of Science, Chinese National Knowledge Infrastructure, Chinese Biomedical Literature Database, Chinese Scientific Journal Database, and Wanfang Database. The search included studies published from the inception of these databases until November 16, 2022 and only randomized controlled trials (RCTs) were considered. To broaden the search, we used a combination of Medical Subject Headings (MeSH) and free text terms, merged using Boolean logical operators as detailed below: “cognitive dysfunction,” “mild cognitive impairment,” “traditional Chinese medicine,” “Chinese patent medicine,” “donepezil,” and “randomized controlled trial.” The search strategies used are presented in detail in Supplementary Table 1. To ensure comprehensive coverage, we conducted additional searches for relevant studies using various sources, including similar systematic reviews and meta-analyses, grey literature such as noncommercial dissertations and government documents, and relevant journals such as Frontiers in Neuroscience, Nature Neuroscience, Chinese Journal of Neurology and Chinese Journal of Nervous and Mental Diseases, as well as major conferences.

### 2.2. Eligibility criteria and literature screen

Eligibility criteria were developed based on the PICOS principle (participants, interventions, comparators, outcomes, and study design).

1. Participants: The inclusion criteria for the studies were limited to participants diagnosed with MCI, whereas studies involving other forms of cognitive dysfunction, such as dementia, were excluded from the analysis.
2. Interventions: The studies considered in this analysis involved the use of donepezil in combination with TCM, specifically Chinese herbal compounds, Chinese patent medicine, single Chinese herbs, and their extracts. The TCM was administered in various forms such as decoction, granule, tablet, capsule, powder, or injection. Studies that solely employed TCM or donepezil in conjunction with non-pharmacological therapies like acupuncture were excluded from this analysis.
3. Comparators: For the purpose of our study, we only considered cases where the control group received a comparable dosage of donepezil. We did not include studies where the control group received no intervention, a placebo, or other medications.
4. Outcomes: This study included primary outcomes related to clinical effectiveness (CE), cognitive assessment tools such as the Mini-mental State Examination (MMSE; Folstein et al., 1975) and the Montreal Cognitive Assessment (MoCA; Nasreddine et al., 2005). Additionally, secondary outcomes were measured using the TCM syndrome scale (TCMSS; Zhuang et al., 2022), the Barthel Index (BI; Mahoney and Barthel, 1965), and AEs.
5. Study design: Our study encompassed all forms of RCTs without limitations on language, region, or publication date. However, we excluded review articles, meta-analyses, case reports, and observational studies from our analysis.

The literature screening process was conducted by two independent authors, with any disagreements resolved by the corresponding author. All studies were managed and screened using EndNote (Version 20.2). Duplicate papers were removed initially, followed by the elimination of irrelevant files through title and abstract browsing. Full-text screening was then conducted on the remaining documents to identify eligible studies.

### 2.3. Data extraction

Based on the Cochrane data extraction criteria (Cumpston et al., 2019), two authors separately extracted key data from the studies, including (1) basic information such as lead author, publication date, region of the trial, etc.; (2) patient characteristics like age, gender, disease course, etc.; (3) treatment methods: the experimental and control groups; (4) literature quality: collect the relevant content based on the Cochrane Risk of Bias (ROB) tool (Cumpston et al., 2019); (5) outcomes: the number of CE and AEs as well as the pre- and post-treatment scores of the MMSE, MoCA, TCMSS, and BI. Any discrepancies were resolved through discussion.

### 2.4. Quality assessment

To evaluate the quality of the studies, the ROB tool (Cumpston et al., 2019) was employed, which consisted of the following seven items: random sequence generation (selection bias), allocation concealment (selection bias), blinding of participants and personnel (performance bias), blinding of outcome assessment (detection bias), incomplete outcome data (attrition bias), selective reporting (reporting bias), and other bias. The level of bias risk for each item was graded as low, unclear or high. For selection bias, we scrutinized the study’s method for generating random sequences and the rigorous assignment of patients with MCI based on random numbers. We also examined whether the study used blind methods for researchers, participants, and evaluators to minimize performance and detection biases. Additionally, we evaluated whether the study had a significant amount of missing data, only reported favorable outcomes, or had other factors that could affect results. The ROB figures were generated using Review Manager (Version 5.4), and two authors independently evaluated the ROB of the included studies. Any discrepancies were settled by the corresponding author.

### 2.5. Statistical analyses

In accordance with the Cochrane Collaboration Handbook (Cumpston et al., 2019), we conducted a conventional paired meta-analysis of the included studies. To calculate the pooled effect size for the dichotomous variable, we utilized relative risk (RR) and 95% confidence interval (CI) while for a continuous variable, we employed weighted mean difference (WMD) and 95% CI. We assessed heterogeneity among the studies using the Cochran *Q*-test and *I*^2^ statistic (Cumpston et al., 2019). If the *p*-value was greater than 0.1 for the *Q*-test and the *I*^2^ statistic was less than 50%, indicating acceptable heterogeneity, we reported the fixed effect model. Otherwise, we reported the random effect model (Higgins and Thompson, 2002; Higgins et al., 2003). To account for potential publication bias, we evaluated the funnel plot for asymmetry and the *p* value of less than 0.05 (Egger et al., 1997, 2003). To investigate the impact of various factors on results and possible sources of heterogeneity, we divided the data into seven subgroups before conducting statistical analyses. These subgroups were based on intervention duration (< 24 weeks vs. ≥ 24 weeks), region (developed vs. undeveloped), publication year (≤ 2018 vs. > 2018), total sample size (< 100 vs. ≥ 100), male to female ratio (≥ 1 vs. < 1), pathogenesis (MCI caused by Parkinson’s disease [MCI-PD] vs. MCI resulting from cerebrovascular disease [MCI-CVD] vs. MCI due to vascular risk factors [MCI-VRF]), and disease course (≤ 2 years vs. > 2 years). To ensure the reliability of our results, we conducted sensitivity analyses by excluding one study at a time. The statistical analyses were carried out using STATA (Version 14.0).

## 3. Results

### 3.1. Literature selection and characteristics of the included studies

**Figure 1:**
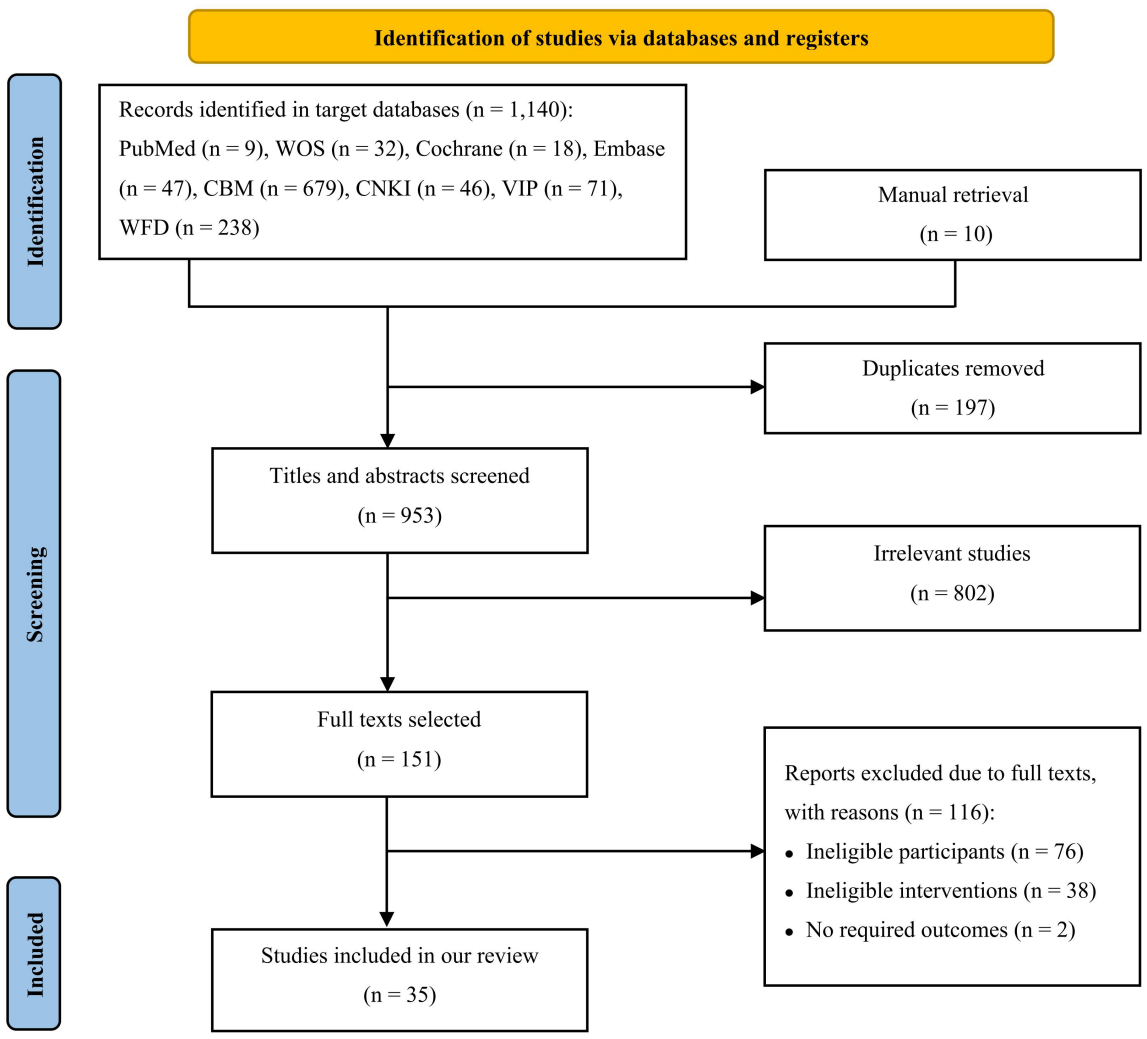
Flowchart of literature selection (CBM: Chinese Biomedical Literature Database; CNKI: Chinese National Knowledge Infrastructure; VIP: Chinese Scientific Journal Database; WFD: Wanfang Database; WOS: Web of Science).

In this study, we collected 1,150 papers, out of which 1,140 were from target databases and 10 were manually retrieved. After eliminating 197 duplicates and 802 irrelevant articles based on title and abstract screening, we reviewed the full text of the remaining 151 articles. Of these, 116 studies were excluded because they were mixed with other types of cognitive dysfunction (76 items), only used TCM or donepezil combined with acupuncture as interventions (38 items), or lacked appropriate outcomes (2 items). Finally, we included 35 RCTs in our review (Liu et al., 2008; Yang et al., 2011; Li and Zhou, 2014; Shen, 2014; Wang et al., 2014; Ye and Feng, 2014; Dong, 2015; Guan et al., 2015; Liu, 2015; Liu and Liu, 2015; Su et al., 2015; Xie and Chen, 2015; Yu, 2016; Di et al., 2017; Gao et al., 2017; He et al., 2017; Gu, 2018; Wang et al., 2018; Luo, 2019; Qian, 2019; Yang, 2019; Gan et al., 2020; Han, 2020; Li and Wang, 2020; Liu, 2020; Zhang, 2020; Wang X. et al., 2021; Yang, 2021; Zhang et al., 2021; Zhou and Du, 2021; Li, 2022; Li et al., 2022; Shou et al., 2022; Wen, 2022; Zhi, 2022). The study selection process is detailed in Figure 1. All RCTs were conducted in China between 2008 and 2022, with a total of 2,833 participants enrolled. The mean age of participants ranged from 51.17 (3.98) to 80.05 (3.40) years, with a lower proportion of female patients (44.18%) than male patients (55.82%; one study was not reported). The number of participants in each study ranged from 30 to 126 and the intervention duration varied from 4 weeks to 12 months. Table 1 provides a summary of the characteristics of the included studies.

**Table 1:** Characteristics of the included studies.

Publications	Sample size		Age (mean ± SD/range)		Proportion of male (%)	Intervention duration	Outcomes
	EG	CG	EG	CG			
Yang et al. (2011)	33	32	65.50 ± 10.90	63.50 ± 8.90	60.29%	60 days	②③⑥
Gu (2018)	35	35	68.40 ± 3.50	69.20 ± 3.70	55.71%	12 weeks	②④⑥
Han (2020)	41	43	56.77 ± 4.67	67.63 ± 3.86	46.43%	12 weeks	①②④⑥
Gao et al. (2017)	60	60	62.40 ± 5.95	62.08 ± 5.92	58.33%	6 months	①②③⑥
Xie and Chen (2015)	30	30	59.12 ± 16.76	60.15 ± 14.68	58.33%	60 days	①②③⑤
Zhang (2020)	63	50	63.70 ± 6.50	63.10 ± 7.30	48.67%	8 weeks	②③⑤⑥
Guan et al. (2015)	52	52	59.00 ± 5.70	60.00 ± 5.20	59.62%	3 months	①②③
Gan et al. (2020)	26	26	57.00 ± 3.40	58.00 ± 2.60	59.62%	12 weeks	②⑥
Wang et al. (2014)	40	32	55.60 ± 4.52	51.17 ± 3.98	59.72%	12 weeks	①②
Yang (2021)	25	34	67.68 ± 10.98	64.50 ± 12.23	59.32%	2 months	②③④⑤⑥
Li and Wang (2020)	30	30	61.50 ± 11.50	62.30 ± 7.80	56.67%	4 weeks	①②
Li and Zhou (2014)	34	34	57.36 ± 9.48	58.46 ± 9.18	57.35%	180 days	②③
Zhi (2022)	30	30	64.70 ± 6.99	63.87 ± 8.05	48.33%	60 days	①②③④⑥
Zhang et al. (2021)	46	45	63.28 ± 10.36	65.47 ± 10.29	52.75%	12 weeks	①②③④⑥
Shou et al. (2022)	41	41	67.90 ± 7.61	68.03 ± 7.77	65.85%	12 weeks	①③⑤
Di et al. (2017)	33	33	71.40 ± 8.80	70.80 ± 8.40	42.42%	12 months	①②⑥
Liu (2015)	50	50	71.05 ± 6.83	72.32 ± 5.30	56.00%	9 months	①②
Liu (2020)	43	43	69.01 ± 3.35	68.36 ± 3.54	50.00%	2 months	①②③④⑤
Liu and Liu (2015)	60	60	64.50 ± 3.00	65.20 ± 3.30	54.17%	9 months	②
Dong (2015)	57	57	66.20 ± 2.60	65.40 ± 2.50	57.89%	60 days	①②⑤
Liu et al. (2008)	16	14	64.92 ± 9.15	67.76 ± 9.13	50.00%	16 weeks	②⑥
Li et al. (2022)	32	32	59.03 ± 7.15	60.06 ± 6.58	67.19%	8 weeks	①②③④⑥
He et al. (2017)	40	40	72.32 ± 5.30	71.05 ± 6.83	56.25%	8 weeks	①②④⑥
Yu (2016)	40	40	NR	NR	NR	2 months	②③⑤
Ye and Feng (2014)	40	36	58–75		51.32%	12 weeks	②⑥
Luo (2019)	30	30	68.20 ± 29.26	66.97 ± 10.51	55.00%	3 months	①②③④⑥
Li et al. (2022)	63	63	62.73 ± 5.12	61.46 ± 6.17	53.17%	8 weeks	①②③⑥
Shen (2014)	38	38	70.32 ± 4.57		65.79%	3 months	②⑥
Su et al. (2015)	50	50	53.50 ± 8.50	51.80 ± 9.60	56.00%	56 days	③
Wang et al. (2018)	45	45	71.20 ± 4.60	70.20 ± 4.20	50.00%	3 months	②
Zhou and Du (2021)	63	63	65.80 ± 2.60	63.90 ± 2.30	53.97%	12 weeks	②③⑥
Wen (2022)	38	38	79.58 ± 4.18	80.05 ± 3.40	68.42%	8 weeks	①②③⑥
Yang (2019)	40	40	64.72 ± 7.55	63.48 ± 7.32	55.00%	12 weeks	①②③⑥
Wang X. et al. (2021)	30	30	68.83 ± 5.50	68.53 ± 5.29	55.00%	3 months	①④⑥
Qian (2019)	31	32	66.13 ± 5.51	67.59 ± 5.30	58.73%	12 weeks	①②③④⑥

CG, control group; EG, experimental group; NR, no reported; Outcomes (① CE, clinical effectiveness; ② MMSE, Mini-mental State Examination; ③ MoCA, Montreal Cognitive Assessment; ④ TCMSS, traditional Chinese medicine syndrome scale; ⑤ BI, Barthel Index; ⑥ AEs, adverse events); SD, standard deviation.

### 3.2. Quality of the included studies

**Figure 2:**
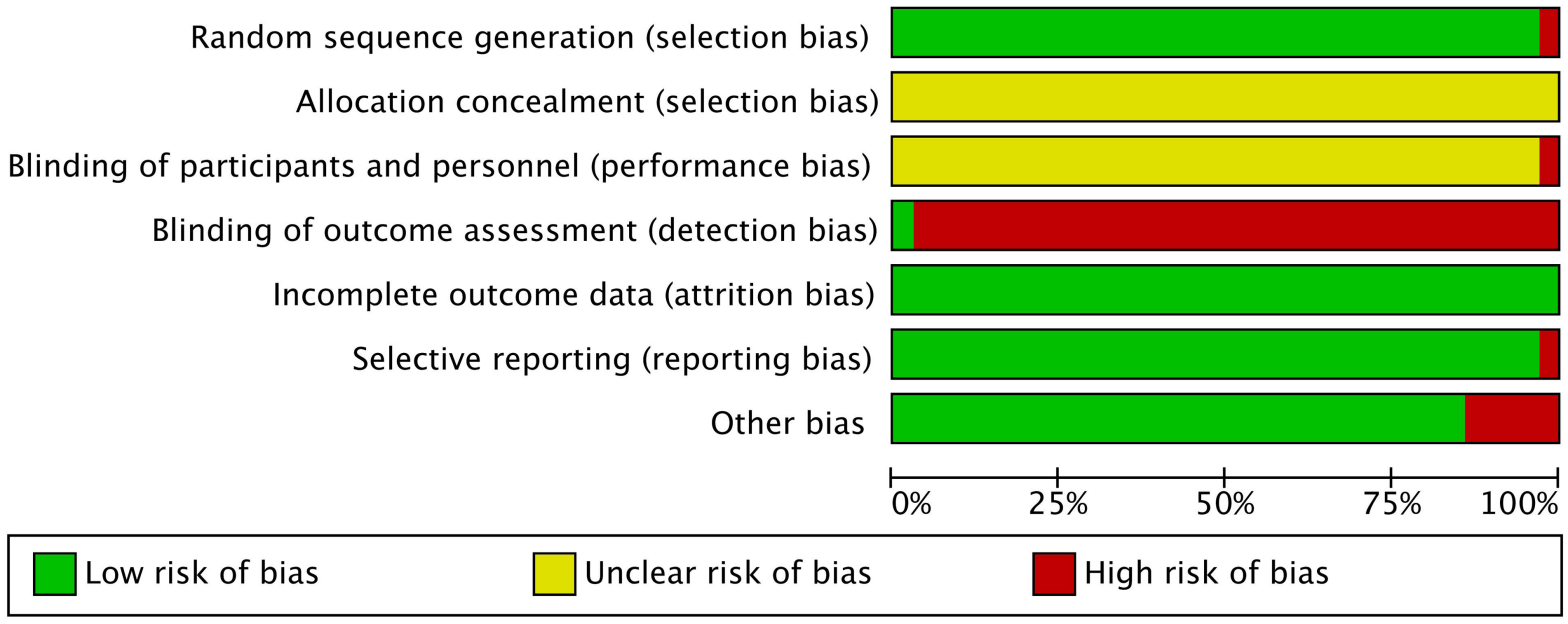
Risk of bias for summary quality (Each item presented as percentages).

This study examines the quality of various studies on random sequence generation, and most studies had low risk except for high risk study conducted by Zhang (2020). Allocation concealment was unclear risk across all studies, while performance bias was mostly unclear risk except for one (Xie and Chen, 2015) high risk. Detection bias was mostly high risk, with only one (Yang, 2021) study judged as low risk. Attrition bias risk was low in all studies, and reporting bias was mostly low risk, except for one high risk study (Yang et al., 2011). The majority of trials had low risk when considering other bias, except for five high risk studies (Shen, 2014; Ye and Feng, 2014; Liu, 2015; Liu and Liu, 2015; Yu, 2016). Figure 2 and Supplementary Figure 1 provide detailed quality assessment results.

### 3.3. Primary outcomes

**Figure 3:**
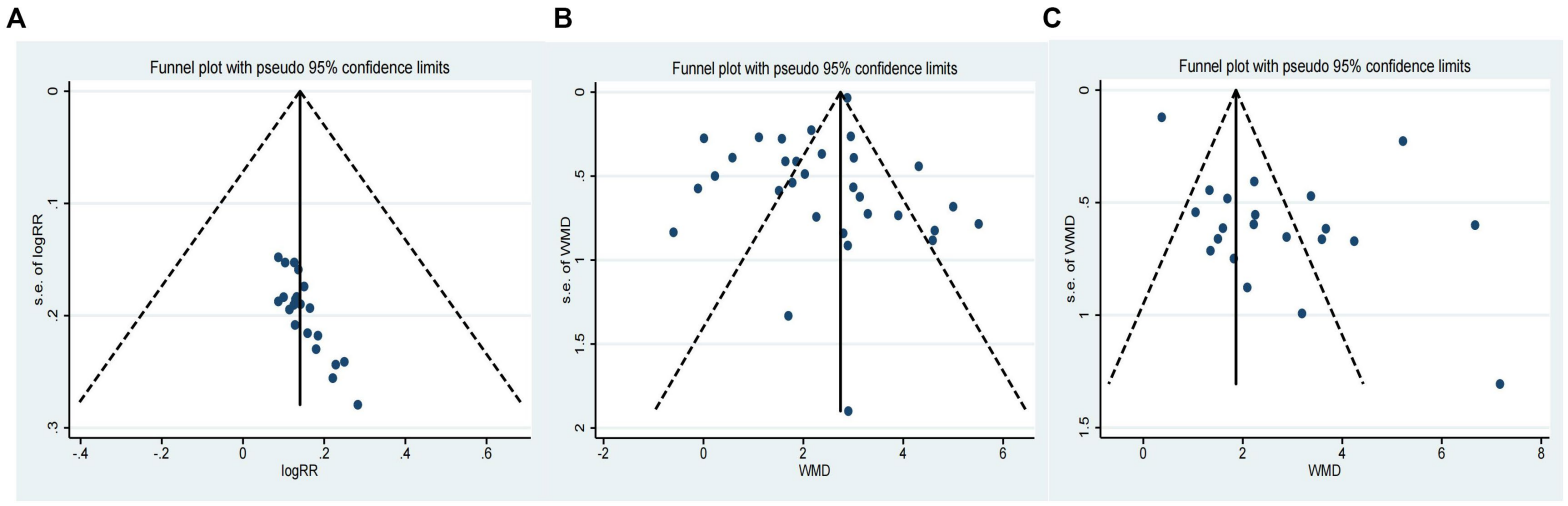
Funnel plot of primary outcomes [**(A)**: Funnel plot of CE; **(B)** Funnel plot of MMSE; **(C)** Funnel plot of MoCA].

The effectiveness of combining donepezil with TCM on CE was evaluated in 21 studies involving 1,708 participants (Wang et al., 2014; Dong, 2015; Guan et al., 2015; Liu, 2015; Xie and Chen, 2015; Di et al., 2017; Gao et al., 2017; He et al., 2017; Luo, 2019; Qian, 2019; Yang, 2019; Han, 2020; Li and Wang, 2020; Liu, 2020; Wang X. et al., 2021; Zhang et al., 2021; Li, 2022; Li et al., 2022; Shou et al., 2022; Wen, 2022; Zhi, 2022). The results showed that donepezil combined with TCM significantly increased CE compared to donepezil alone (RR = 1.15, 95% CI: 1.06 to 1.25, *p* > 0.999, *I*^2^ = 0.00%, fixed model; as shown in Table 2). However, the asymmetrical funnel plot (refer to Figure 3) and Egger’s test (*p* < 0.05) suggested potential publication bias.

**Table 2:** Outcomes and subgroup analyses based on primary outcomes.

Meta-analyses variables	Number of studies	Number of patients		Pooled effect sizes	Heterogeneity	
		EG	CG		*p*	*I*^2^ (%)
*Dichotomous variables*				RR (95% CI)		
CE	21	857	851	1.15 (1.06 to 1.25)	> 0.999	0.00%
AEs	16	608	593	0.86 (0.62 to 1.19)	0.844	0.00%
*Continuous variables*				WMD (95% CI)		
MMSE	31	1,278	1,261	2.33 (1.90 to 2.76)	< 0.001	91.40%
MoCA	21	870	865	2.79 (1.82 to 3.75)	< 0.001	96.00%
TCMSS	11	383	394	−3.01 (−3.79 to −2.23)	< 0.001	81.10%
BI	7	299	295	9.20 (5.39 to 13.00)	< 0.001	83.40%
*Subgroup analyses based on CE*				RR (95% CI)		
*Intervention duration*						
Overall	21	857	851	1.15 (1.06 to 1.25)	> 0.999	0.00%
< 24 weeks	18	714	708	1.15 (1.05 to 1.26)	> 0.999	0.00%
≥ 24 weeks	3	143	143	1.15 (0.95 to 1.40)	0.975	0.00%
*Region*						
Overall	21	857	851	1.15 (1.06 to 1.25)	> 0.999	0.00%
Developed	8	320	320	1.18 (1.03 to 1.36)	0.999	0.00%
Undeveloped	13	537	531	1.14 (1.03 to 1.26)	> 0.999	0.00%
*Publication year*						
Overall	21	857	851	1.15 (1.06 to 1.25)	> 0.999	0.00%
≤ 2018	8	362	354	1.15 (1.01 to 1.30)	> 0.999	0.00%
> 2018	13	495	497	1.16 (1.04 to 1.29)	> 0.999	0.00%
*Total sample size*						
Overall	21	857	851	1.15 (1.06 to 1.25)	> 0.999	0.00%
< 100	16	575	569	1.17 (1.05 to 1.29)	> 0.999	0.00%
≥ 100	5	282	282	1.13 (0.98 to 1.30)	0.998	0.00%
*Male to female ratio*						
Overall	21	857	851	1.15 (1.06 to 1.25)	> 0.999	0.00%
≥ 1	19	783	775	1.15 (1.06 to 1.25)	> 0.999	0.00%
< 1	2	74	76	1.17 (0.88 to 1.54)	0.846	0.00%
*Pathogenesis*						
Overall	11	472	465	1.14 (1.02 to 1.28)	> 0.999	0.00%
MCI-PD	4	154	153	1.15 (0.95 to 1.39)	0.953	0.00%
MCI-CVD	4	163	157	1.17 (0.96 to 1.42)	0.984	0.00%
MCI-VRF	3	155	155	1.12 (0.93 to 1.34)	0.979	0.00%
*Disease course*						
Overall	11	462	463	1.15 (1.03 to 1.29)	> 0.999	0.00%
≤ 2 years	6	263	265	1.14 (0.99 to 1.33)	> 0.999	0.00%
> 2 years	5	199	198	1.16 (0.98 to 1.37)	0.999	0.00%
*Subgroup analyses based on MMSE*				WMD (95% CI)		
*Intervention duration*						
Overall	31	1,278	1,261	2.33 (1.90 to 2.76)	< 0.001	91.40%
< 24 weeks	26	1,041	1,024	2.16 (1.70 to 2.62)	< 0.001	91.00%
≥ 24 weeks	5	237	237	3.33 (1.64 to 5.02)	< 0.001	93.10%
*Region*						
Overall	29	1,210	1,194	2.39 (1.95 to 2.83)	< 0.001	91.30%
Developed	11	461	461	2.33 (1.76 to 2.90)	< 0.001	88.50%
Undeveloped	18	749	733	2.50 (1.81 to 3.19)	< 0.001	89.10%
*Publication year*						
Overall	31	1,278	1,261	2.33 (1.90 to 2.76)	< 0.001	91.40%
≤ 2018	17	703	688	2.40 (1.74 to 3.07)	< 0.001	90.20%
> 2018	14	575	573	2.26 (1.64 to 2.88)	< 0.001	88.70%
*Total sample size*						
Overall	31	1,278	1,261	2.33 (1.90 to 2.76)	< 0.001	91.40%
< 100	23	810	806	1.74 (1.29 to 2.18)	< 0.001	80.50%
≥ 100	8	468	455	3.77 (3.18 to 4.36)	< 0.001	81.30%
*Male to female ratio*						
Overall	30	1,238	1,221	2.30 (1.86 to 2.74)	< 0.001	91.60%
≥ 1	27	1,101	1,095	2.32 (1.85 to 2.78)	< 0.001	90.90%
< 1	3	137	126	2.12 (0.91 to 3.34)	< 0.001	87.90%
*Pathogenesis*						
Overall	15	673	649	2.54 (2.01 to 3.06)	< 0.001	87.40%
MCI-PD	5	217	216	2.78 (2.20 to 3.35)	0.079	52.20%
MCI-CVD	6	266	243	1.91 (0.73 to 3.09)	< 0.001	94.30%
MCI-VRF	4	190	190	3.22 (1.86 to 4.59)	0.002	80.50%
*Disease course*						
Overall	14	628	616	2.78 (2.22 to 3.34)	< 0.001	83.80%
≤ 2 years	8	357	359	2.63 (1.84 to 3.42)	< 0.001	89.80%
> 2 years	6	271	257	3.00 (2.15 to 3.84)	0.045	55.90%
*Subgroup analyses based on MoCA*				WMD (95% CI)		
*Intervention duration*						
Overall	21	870	865	2.79 (1.82 to 3.75)	< 0.001	96.00%
< 24 weeks	19	776	771	2.50 (1.54 to 3.46)	< 0.001	95.70%
≥ 24 weeks	2	94	94	5.47 (3.09 to 7.85)	0.007	86.30%
*Region*						
Overall	20	837	833	2.88 (1.87 to 3.88)	< 0.001	96.20%
Developed	10	393	393	2.50 (0.93 to 4.06)	< 0.001	97.70%
Undeveloped	10	444	440	3.22 (2.12 to 4.32)	< 0.001	87.50%
*Publication year*						
Overall	21	870	865	2.79 (1.82 to 3.75)	< 0.001	96.00%
≤ 2018	7	299	298	3.35 (1.33 to 5.37)	< 0.001	96.50%
> 2018	14	571	567	2.54 (1.65 to 3.43)	< 0.001	90.80%
*Total sample size*						
Overall	21	870	865	2.79 (1.82 to 3.75)	< 0.001	96.00%
< 100	15	519	527	2.30 (1.78 to 2.81)	< 0.001	64.70%
≥ 100	6	351	338	4.07 (1.52 to 6.62)	< 0.001	98.90%
*Male to female ratio*						
Overall	20	830	825	2.78 (1.79 to 3.78)	< 0.001	96.20%
≥ 1	19	767	775	2.76 (1.74 to 3.79)	< 0.001	96.40%
< 1	1	63	50	3.19 (1.24 to 5.14)	/	/
*Pathogenesis*						
Overall	12	531	518	2.86 (1.42 to 4.30)	< 0.001	97.60%
MCI-PD	5	218	218	3.39 (2.02 to 4.76)	< 0.001	91.50%
MCI-CVD	3	145	132	1.47 (−0.02 to 2.96)	0.003	82.70%
MCI-VRF	4	168	168	3.14 (0.79 to 5.49)	< 0.001	93.60%
*Disease course*						
Overall	8	341	327	2.23 (1.05 to 3.42)	< 0.001	90.40%
≤ 2 years	4	159	159	2.46 (0.30 to 4.61)	< 0.001	94.60%
> 2 years	4	182	168	1.95 (1.28 to 2.63)	0.388	0.80%

AEs, adverse events; BI, Barthel Index; CE, clinical effectiveness; CG, control group; CI, confidence interval; EG, experimental group; MCI-PD, mild cognitive impairment caused by Parkinson’s disease; MCI-CVD, mild cognitive impairment resulting from cerebrovascular disease; MCI-VRF, mild cognitive impairment due to vascular risk factors; MMSE, Mini-mental State Examination; MoCA, Montreal Cognitive Assessment; RR, relative risk; TCMSS, traditional Chinese medicine syndrome scale; WMD, weighted mean difference.

The cognitive function of 2,539 participants was assessed in 31 studies using the MMSE (Liu et al., 2008; Yang et al., 2011; Li and Zhou, 2014; Shen, 2014; Wang et al., 2014; Ye and Feng, 2014; Dong, 2015; Guan et al., 2015; Liu, 2015; Liu and Liu, 2015; Xie and Chen, 2015; Yu, 2016; Di et al., 2017; Gao et al., 2017; He et al., 2017; Gu, 2018; Wang et al., 2018; Luo, 2019; Qian, 2019; Yang, 2019; Han, 2020; Li and Wang, 2020; Liu, 2020; Zhang, 2020; Yang, 2021; Zhang et al., 2021; Zhou and Du, 2021; Li, 2022; Li et al., 2022; Wen, 2022; Zhi, 2022). The results showed that donepezil combined with TCM was more effective than donepezil in promoting cognitive function recovery (WMD = 2.33, 95% CI: 1.90 to 2.76, *p* < 0.001, *I*^2^ = 91.40%, random model; as shown in Table 2). The symmetrical funnel plot (refer to Figure 3) and Egger’s test (*p* > 0.05) indicated no apparent publication bias.

In 21 studies involving 1,735 participants, cognitive function was evaluated using the MoCA (Yang et al., 2011; Li and Zhou, 2014; Guan et al., 2015; Su et al., 2015; Xie and Chen, 2015; Yu, 2016; Gao et al., 2017; Luo, 2019; Qian, 2019; Yang, 2019; Gan et al., 2020; Liu, 2020; Zhang, 2020; Yang, 2021; Zhang et al., 2021; Zhou and Du, 2021; Li, 2022; Li et al., 2022; Shou et al., 2022; Wen, 2022; Zhi, 2022). Results indicated that the combination of donepezil and TCM led to a significant improvement in cognition when compared to donepezil alone (WMD = 2.79, 95% CI: 1.82 to 3.75, *p* < 0.001, *I*^2^ = 96.00%, random model; as shown in Table 2). However, the asymmetrical funnel plot (refer to Figure 3) and Egger’s test (*p* < 0.05) suggested the possibility of publication bias.

### 3.4. Secondary outcomes

In 11 trials involving 777 participants, the efficacy of TCMSS was studied (He et al., 2017; Gu, 2018; Luo, 2019; Qian, 2019; Han, 2020; Liu, 2020; Wang X. et al., 2021; Yang, 2021; Zhang et al., 2021; Li et al., 2022; Zhi, 2022). The results indicated that the combination of donepezil and TCM was effective in alleviating symptoms of Chinese medicine as compared to donepezil alone (WMD = −3.01, 95% CI: −3.79 to −2.23, *p* < 0.001, *I*^2^ = 81.10%, random model; as shown in Table 2). Although the funnel plot showed asymmetry (refer to Supplementary Figure 2), the Egger’s test (*p* > 0.05) indicated no significant publication bias.

In 7 trials involving 594 participants, the BI was utilized to assess activities of daily living (Dong, 2015; Xie and Chen, 2015; Yu, 2016; Liu, 2020; Zhang, 2020; Yang, 2021; Shou et al., 2022). Our study found that when donepezil was combined with TCM, it significantly improved activities of daily living compared to donepezil alone (WMD = 9.20, 95% CI: 5.39 to 13.00, *p* < 0.001, *I*^2^ = 83.40%, random model; as shown in Table 2). However, potential publication bias was suggested by the asymmetry of the funnel plot (refer to Supplementary Figure 2) and confirmed by the Egger’s test (*p* < 0.05).

In 16 trials involving 1,201 participants (Liu et al., 2008; Yang et al., 2011; Shen, 2014; Di et al., 2017; Gao et al., 2017; He et al., 2017; Gu, 2018; Luo, 2019; Qian, 2019; Yang, 2019; Gan et al., 2020; Zhang, 2020; Wang X. et al., 2021; Zhou and Du, 2021; Li et al., 2022; Wen, 2022), it was reported that the combination of donepezil and TCM did not significantly reduce the incidence of AEs compared to donepezil alone (RR = 0.86, 95% CI: 0.62 to 1.19, P = 0.844, I2 = 0.00%, fixed model; as shown in Table 2). The funnel plot (refer to Supplementary Figure 2) and Egger’s test (*p* > 0.05) both indicated no evidence of publication bias.

### 3.5. Subgroup analyses and sensitivity analyses

Subgroup analyses of primary outcomes were conducted and the results showed that the majority of subgroups had consistent results. There was no significant difference observed between each subitem within the subgroup. However, in subgroup analyses of CE, studies with an intervention duration <24 weeks (RR = 1.15, 95% CI: 1.05 to 1.26, *p* > 0.999, *I*^2^ = 0.00%, fixed model), a total sample size <100 (RR = 1.17, 95% CI: 1.05 to 1.29, *p* > 0.999, *I*^2^ = 0.00%, fixed model), and a male to female ratio ≥ 1 (RR = 1.15, 95% CI: 1.06 to 1.25, *p* > 0.999, *I*^2^ = 0.00%, fixed model) showed a significant improvement in CE compared to studies with an intervention duration ≥24 weeks (RR = 1.15, 95% CI: 0.95 to 1.40, *p* = 0.975, *I*^2^ = 0.00%, fixed model), or more, a total sample size ≥100 (RR = 1.13, 95% CI: 0.98 to 1.30, *p* = 0.998, *I*^2^ = 0.00%, fixed model), or more, a male to female ratio < 1 (RR = 1.17, 95% CI: 0.88 to 1.54, *p* = 0.846, *I*^2^ = 0.00%, fixed model). In subgroup analyses of MoCA, it was observed that studies with disease course >2 years (WMD = 1.95, 95% CI: 1.28 to 2.63, *p* = 0.388, *I*^2^ = 0.80%, random model) had less heterogeneity compared to studies with disease course ≤2 years (WMD = 2.46, 95% CI: 0.30 to 4.61, *p* < 0.001, *I*^2^ = 94.60%, random model). In addition, the MoCA score showed a significant increase in patients with MCI-PD (WMD = 3.39, 95% CI: 2.02 to 4.76, *p* < 0.001, *I*^2^ = 91.50%, random model) and MCI-VRF (WMD = 3.14, 95% CI: 0.79 to 5.49, *p* < 0.001, *I*^2^ = 93.60%, random model), but not in those with MCI-CVD (WMD = 1.47, 95% CI: −0.02 to 2.96, *p* = 0.003, *I*^2^ = 82.70%, random model), when studies were classified by pathogenesis. Table 2 lists the detailed subgroup analyses. The sensitivity analyses suggested that all findings were robust.

## 4. Discussion

This study is the first to systematically review and analyze the effectiveness and safety of combining donepezil and TCM for patients with MCI. An extensive literature search was conducted based on eight electronic databases and manual retrieval, ultimately identifying 35 studies with a total of 2,833 participants. Our results showed that combing donepezil and TCM significantly improved cognitive function and daily activities compared to donepezil alone. In addition, patients with MCI-PD and MCI-VRF showed a significant improvement in cognitive function, but this benefit was not observed in patients with MCI-CVD.

The results of our efficacy study revealed that the combination of donepezil and TCM led to a significant improvement in MMSE, MoCA, and BI scores compared to donepezil alone. This suggests that the combined treatment may be more effective in enhancing cognitive function and daily activities. Our findings align with previous meta-analyses conducted on the use of WM combined with TCM or TCM alone for patients with MCI. A meta-analysis of 21 RCTs demonstrated that the combination of WM and *Ginkgo Biloba* was more effective in improving cognitive function than WM alone (Yang et al., 2016). Another meta-analysis with 1,683 patients with MCI found that TCM significantly improved cognitive function and activities of daily living compared to no intervention and a placebo (Liang et al., 2022). Numerous scholars have conducted in-depth research to explore the intrinsic mechanism of TCM in cognition and have observed remarkable benefits. One such study (Zhang H. et al., 2022) observed that the Guilingji capsule increased serum levels of acetylcholine (Ach) while decreasing serum levels of acetylcholinesterase (AchE), homocysteine (Hcy), and high-sensitivity C-reactive protein (hs-CRP) in patients with MCI. This suggests that the mechanism for improving cognition may be related to regulating the cholinergic system and suppressing inflammation. According to a study (Han et al., 2014), Qingnao Yizhi granules were found to be effective in increasing serum levels of superoxide dismutase (SOD) and decreasing serum levels of malondialdehyde (MDA) and AchE in patients with MCI. This suggests that the Qingnao Yizhi granules may enhance cognitive function by scavenging free radicals, inhibiting brain tissue peroxidation, and promoting brain cell metabolism. Additionally, the study (Huang et al., 2022) found that serum levels of uric acid (UA) and SOD were significantly positively correlated with the MoCA score, indicating a potential relationship between these factors and cognitive function in patients with MCI. Research has shown that the steroid-enriched fraction of *Achyranthes bidentata* Blume can reduce oxidative stress and neuroinflammatory response in the cortical and hippocampal regions by modulating the MAPKs/NF-κB signaling pathway, leading to improved cognitive function (Lin et al., 2019). In addition, senegenin can exhibit anti-inflammatory, antioxidant, anti-apoptotic, and neurotrophic activity by regulating various pathways such as MAPK/NF-κB, Nrf2/HO-1, PI3K/Akt, and ROS/Ca^2+^. These findings suggest that senegenin has neuroprotective effects (Chen et al., 2022).

The safety of combining donepezil with TCM is a crucial consideration. Our results found no significant difference in safety between donepezil combined with TCM and donepezil alone. During the intervention, a small number of participants in both groups experienced AEs, such as gastrointestinal symptoms, sleep disturbances, fatigue, dizziness, and muscle cramps. Most of these AEs were mild in intensity and consistent with those reported in previous studies on donepezil (Zhang X. et al., 2022). Previous studies have shown that TCM is relatively safe and there was no significant difference in AEs between TCM and a placebo (Zhang et al., 2019). In this review, we focused on the AEs caused by donepezil and investigated the correlation between the safety of donepezil and dosage forms, doses, and intervention durations. Firstly, our findings suggest that donepezil transdermal patches have less gastrointestinal harm compared to oral administration. The most common AEs reported among the studies included gastrointestinal symptoms, such as nausea, vomiting, diarrhea, constipation, gastric distention, and decreased appetite. One study (Larkin, 2022) suggested that donepezil transdermal patches could reduce gastrointestinal damage and frequency of administration compared to the oral route, improving medication compliance and alleviating AEs. However, all participants in previous studies took donepezil orally. Therefore, more large sample sizes, multi-center, and high-quality RCTs should be conducted in the future to validate the safety of donepezil transdermal patches combined with TCM for patients with MCI. According to studies, donepezil is safer in smaller doses. In fact, patients who took 10 mg/day had a higher risk of AEs and early withdrawal from the trial compared to those who received 5 mg/day (Birks and Harvey, 2018). On the other hand, another study (Battle et al., 2021) found no significant difference in AEs between 5 mg/day of donepezil and a placebo. Based on the above conclusions, it is believed that donepezil at a dose of 5 mg/day is relatively safe for patients with MCI. To minimize harm, we recommend titration administration starting at 5 mg/day of donepezil for 4 weeks before increasing to the necessary 10 mg/day dose (Homma et al., 2016). Additionally, a 3-year study (Winblad et al., 2006) demonstrated a decrease in the incidence of donepezil-related AEs over time. Therefore, we concluded that long-term intervention with donepezil is well-tolerated and early AEs can be managed by adjusting the dosage forms and doses.

In subgroup analyses of pathogenesis, our results showed that patients with MCI-PD and MCI-VRF experienced a significant increase in both the MoCA and MMSE scores, while patients with MCI-CVD only showed a significant enhancement in the MMSE score. Although both scales are commonly used scales to assess cognitive function, the MoCA is more sensitive for patients with MCI (Liang et al., 2023). Therefore, we concluded that the combination of donepezil and TCM can improve cognitive function in patients with MCI-PD and MCI-VRF, but the benefit may be limited in patients with MCI-CVD. A previous study (Liang et al., 2022) has a significant positive effect on the MoCA score in patients with vascular-MCI. To further explore this, we subdivided the vascular-MCI into two subtypes: MCI-VRF and MCI-CVD. This was done because MCI-VRF which does not show visible cerebrovascular lesions on conventional imaging may be an early stage before MCI-CVD (Jia et al., 2014). Our findings confirmed that patients with MCI-VRF showed a significant cognitive improvement compared to patients with MCI-CVD, highlighting the importance of early intervention.

### 4.1. Strengths and limitations

Previous studies on the combination of donepezil and TCM for patients with MCI were limited by small sample sizes and single-center studies, which hindered the generalizability of their conclusions to clinical practice. In contrast, our study provides a comprehensive analysis of previous data, which quantifies the efficacy and safety of this combination. This strengthens the evidence-based medical evidence available for clinical practice and policy development. Following the PICOS principle, we established strict criteria for inclusion and exclusion to maintain consistency among the studies. This approach improves the reliability and validity of our findings. In contrast to earlier studies, we explored a broader range of outcome measures (CE, MMSE, MoCA, TCMSS, BI, and AEs), which provides a comprehensive understanding of the advantages of combining donepezil with TCM in patients with MCI. Seven subgroups were created based on disease characteristics to investigate the impact of different factors on primary outcomes and potential sources of heterogeneity. Our study delved deeper than previous studies and revealed that patients with MCI-VRF experienced more significant cognitive improvement than those with MCI-VRF. We recommend conducting more high-quality RCTs in the future to confirm this discovery. Given that MCI-VRF is an earlier manifestation of MCI-CVD, early intervention may offer greater opportunities for patients with MCI. This has significant implications for clinical science decision-making and policy-making. To address the limited number of RCTs in previous studies, we performed an extensive search of larger electronic databases and supplemented manual retrieval to obtain more eligible literature. As a result, our study was performed on larger total sample sizes, making our findings more reliable than previous conclusions.

This study has some limitations that need to be taken into account when interpreting the results. Firstly, most of the studies included in this review had a high risk of detection bias, which could affect the precision of the findings and consequently the overall quality of our study. Secondly, although we did not include participants with diverse backgrounds, heterogeneity remained high. However, we believe that this heterogeneity may be objective and inevitable due to the population with MCI being in a transitional state between normal aging and early dementia. Therefore, caution should be exercised when interpreting the results of this study.

## 5. Conclusion

According to our study, the combination of donepezil and TCM may have a greater positive impact on cognitive function and daily activities compared to using donepezil alone. However, there was no significant difference in terms of safety. To strengthen our findings and provide more reliable evidence-based medical evidence, it is recommended that more high-quality RCTs be conducted in the future.

## Author contributions

S-jY served as principal author and had full access to all the data in the study, takes responsibility for the accuracy of the data analysis, and the integrity of the data. J-hL and Z-hT contributed to the conception and design. ZL, H-lT, and S-jY contributed to data acquisition and interpretation. S-jY and C-lB contributed to the draft of the manuscript. J-hL, Z-hT, and W-hL contributed to revise of the article and final approval. All authors contributed to the article and approved the submitted version.

## Funding

This research was supported by the National Natural Science Foundation of China (No. 81873204) and the Sichuan Science and Technology Program (Nos. 2021YFS0040 and 2022ZYD0075).

## Conflict of interest

The authors declare that the research was conducted in the absence of any commercial or financial relationships that could be construed as a potential conflict of interest.

## Publisher’s note

All claims expressed in this article are solely those of the authors and do not necessarily represent those of their affiliated organizations, or those of the publisher, the editors and the reviewers. Any product that may be evaluated in this article, or claim that may be made by its manufacturer, is not guaranteed or endorsed by the publisher.

## Supplementary material

Supplementary Figure 1 Risk of bias for individual quality.

Supplementary Figure 2 Funnel plot of secondary outcomes.

Supplementary Figure 3 Flowchart of method implementation.
