# Supplementary material for: Donepezil combined with traditional Chinese medicine has promising efficacy on mild cognitive impairment: a systematic review and meta-analysis
Source: Front Neurosci. 2023 Jul 5;17:1206491. doi: 10.3389/fnins.2023.1206491 (PMC10354366; doi:10.3389/fnins.2023.1206491)
Supplement: Supplementary file 1 [file Table_1.DOCX]

**Supplementary Table 1. Search strategies**

| **PubMed** | | |
| --- | --- | --- |
| Number | Query | Results |
| #1 | "Cognitive Dysfunction"[Mesh] | 32,700 |
| #2 | ((((((((((((Cognitive Dysfunction*[Title/Abstract]) OR (Dysfunction*, Cognitive[Title/Abstract])) OR (Cognitive Impairment*[Title/Abstract])) OR (Impairment*, Cognitive[Title/Abstract])) OR (Mild Cognitive Impairment*[Title/Abstract])) OR (Impairment*, Mild Cognitive[Title/Abstract])) OR (Mild Neurocognitive Disorder*[Title/Abstract])) OR (Disorder*, Mild Neurocognitive[Title/Abstract])) OR (Neurocognitive Disorder*, Mild[Title/Abstract])) OR (Cognitive Decline*[Title/Abstract])) OR (Decline*, Cognitive[Title/Abstract])) OR (Mental Deterioration*[Title/Abstract])) OR (Deterioration*, Mental[Title/Abstract]) | 179,534 |
| #3 | #1 OR #2 | 181,752 |
| #4 | "Medicine, Chinese Traditional"[Mesh] | 22,949 |
| #5 | (((((((((((((Chinese Medicine, Traditional[Title/Abstract]) OR (Chinese Traditional Medicine[Title/Abstract])) OR (Traditional Chinese Medicine[Title/Abstract])) OR (Traditional Medicine, Chinese[Title/Abstract])) OR (Chinese Medicine[Title/Abstract])) OR (TCM[Title/Abstract])) OR ("Chinese and Western Medicine"[Title/Abstract])) OR ("integrated Chinese and Western Medicine"[Title/Abstract])) OR (Chinese herbal[Title/Abstract])) OR (Chinese proprietary medicine[Title/Abstract])) OR (Chinese patent medicine[Title/Abstract])) OR (Decoction*[Title/Abstract])) OR (Chinese medical formula*[Title/Abstract])) OR (Chinese medicine prescription*[Title/Abstract]) | 55,008 |
| #6 | #4 OR #5 | 64,587 |
| #7 | "Donepezil"[Mesh] | 2,803 |
| #8 | ((Donepezil Hydrochloride[Title/Abstract]) OR (Donepezilium Oxalate Trihydrate[Title/Abstract])) OR (Aricept[Title/Abstract]) | 382 |
| #9 | #7 OR #8 | 2,951 |
| #10 | (randomized controlled trial [pt] OR controlled clinical trial [pt] OR randomized [tiab] OR placebo [tiab] OR drug therapy [sh] OR randomly [tiab] OR trial [tiab] OR groups [tiab]) NOT (animals [mh] NOT humans [mh]) | 4,875,136 |
| #11 | #3 AND #16 AND #9 AND #10 | 9 |
| **Embase** | | |
| Number | Query | Results |
| #1 | 'cognitive defect'/exp | 582,673 |
| #2 | 'cognitive dysfunction':ab,ti OR 'cognitive dysfunctions':ab,ti OR 'dysfunction, cognitive':ab,ti OR 'dysfunctions, cognitive':ab,ti OR 'cognitive impairments':ab,ti OR 'cognitive impairment':ab,ti OR 'impairment, cognitive':ab,ti OR 'impairments, cognitive':ab,ti OR 'mild cognitive impairment':ab,ti OR 'cognitive impairment, mild':ab,ti OR 'cognitive impairments, mild':ab,ti OR 'impairment, mild cognitive':ab,ti OR 'impairments, mild cognitive':ab,ti OR 'mild cognitive impairments':ab,ti OR 'mild neurocognitive disorder':ab,ti OR 'disorder, mild neurocognitive':ab,ti OR 'disorders, mild neurocognitive':ab,ti OR 'mild neurocognitive disorders':ab,ti OR 'neurocognitive disorder, mild':ab,ti OR 'neurocognitive disorders, mild':ab,ti OR 'cognitive decline':ab,ti OR 'cognitive declines':ab,ti OR 'decline, cognitive':ab,ti OR 'declines, cognitive':ab,ti OR 'mental deterioration':ab,ti OR 'mental deteriorations':ab,ti OR 'deterioration, mental':ab,ti OR 'deteriorations, mental':ab,ti | 186,524 |
| #3 | #1 OR #2 | 613,786 |
| #4 | 'Chinese medicine'/exp | 67,062 |
| #5 | 'Chinese medicine, traditional':ab,ti OR 'Chinese traditional medicine':ab,ti OR 'traditional Chinese medicine':ab,ti OR 'traditional medicine, Chinese':ab,ti OR 'Chinese medicine':ab,ti OR 'tcm':ab,ti OR 'Chinese and Western medicine':ab,ti OR 'integrated Chinese and Western medicine':ab,ti OR 'Chinese herbal':ab,ti OR 'Chinese proprietary medicine':ab,ti OR 'Chinese patent medicine':ab,ti OR 'decoction':ab,ti OR 'Chinese medical formula':ab,ti OR 'Chinese medicine prescription':ab,ti OR 'traditional Chinese medicine prescription':ab,ti | 75,299 |
| #6 | #4 OR #5 | 100,517 |
| #7 | 'donepezil'/exp | 14,954 |
| #8 | 'donepezil hydrochloride':ab,ti OR 'donepezilium oxalate trihydrate':ab,ti OR 'aricept':ab,ti | 643 |
| #9 | #7 OR #8 | 14,998 |
| #10 | 'crossover procedure':de OR 'double-blind procedure':de OR 'randomized controlled trial':de OR 'single-blind procedure':de OR (random* OR factorial* OR crossover* OR cross NEXT/1 over* OR placebo* OR doubl* NEAR/1 blind* OR singl* NEAR/1 blind* OR assign* OR allocat* OR volunteer*):de,ab,ti | 1,574,261 |
| #11 | #3 AND #16 AND #9 AND #10 | 47 |
| **Cochrane** | | |
| Number | Query | Results |
| #1 | MeSH descriptor: [Cognitive Dysfunction] explode all trees | 2,364 |
| #2 | (Cognitive Dysfunction*):ti,ab,kw OR (Dysfunction*, Cognitive):ti,ab,kw OR (Cognitive Impairment*):ti,ab,kw OR (Impairment*, Cognitive):ti,ab,kw OR (Mild Cognitive Impairment*):ti,ab,kw | 22,413 |
| #3 | (Impairment*, Mild Cognitive):ti,ab,kw OR (Mild Neurocognitive Disorder*):ti,ab,kw OR (Disorder*, Mild Neurocognitive):ti,ab,kw OR (Neurocognitive Disorder*, Mild):ti,ab,kw OR (Cognitive Decline*):ti,ab,kw | 7,058 |
| #4 | (Decline*, Cognitive):ti,ab,kw OR (Mental Deterioration*):ti,ab,kw OR (Deterioration*, Mental):ti,ab,kw | 1,098 |
| #5 | #1 or #2 or #3 or #4 | 27,347 |
| #6 | MeSH descriptor: [Medicine, Chinese Traditional] explode all trees | 1,280 |
| #7 | (Chinese Medicine, Traditional):ti,ab,kw OR (Chinese Traditional Medicine):ti,ab,kw OR (Traditional Chinese Medicine):ti,ab,kw OR (Traditional Medicine, Chinese):ti,ab,kw OR (Chinese Medicine):ti,ab,kw | 14,135 |
| #8 | (TCM):ti,ab,kw OR (Chinese and Western Medicine):ti,ab,kw OR (integrated Chinese and Western Medicine):ti,ab,kw OR (Chinese herbal):ti,ab,kw OR (Chinese proprietary medicine):ti,ab,kw | 13,184 |
| #9 | (Chinese patent medicine):ti,ab,kw OR (Decoction*):ti,ab,kw OR (Chinese medical formula*):ti,ab,kw OR (Chinese medicine prescription*):ti,ab,kw | 5,145 |
| #10 | #6 or #7 or #8 or #9 | 20,888 |
| #11 | MeSH descriptor: [Donepezil] explode all trees | 631 |
| #12 | (Donepezil Hydrochloride):ti,ab,kw OR (Donepezilium Oxalate Trihydrate):ti,ab,kw OR (Aricept):ti,ab,kw | 287 |
| #13 | #11 or #12 | 829 |
| #14 | #5 and #10 and #13 | 18 |
| **Web of Science** | | |
| Number | Query | Results |
| #1 | TS=(Cognitive Dysfunction OR Cognitive Dysfunction* OR Dysfunction*, Cognitive OR Cognitive Impairment* OR Impairment*, Cognitive OR Mild Cognitive Impairment* OR Impairment*, Mild Cognitive OR Mild Neurocognitive Disorder* OR Disorder*, Mild Neurocognitive OR Neurocognitive Disorder*, Mild OR Cognitive Decline* OR Decline*, Cognitive OR Mental Deterioration* OR Deterioration*, Mental) | 168,065 |
| #2 | TS=(Medicine, Chinese Traditional OR Chinese Medicine, Traditional OR Chinese Traditional Medicine OR Traditional Chinese Medicine OR Traditional Medicine, Chinese OR Chinese Medicine OR TCM OR Chinese and Western Medicine OR integrated Chinese and Western Medicine OR Chinese herbal OR Chinese proprietary medicine OR Chinese patent medicine OR Decoction* OR Chinese medical formula* OR Chinese medicine prescription*) | 55,523 |
| #3 | TS=(Donepezil OR Donepezil Hydrochloride OR Donepezilium Oxalate Trihydrate OR Aricept) | 3,897 |
| #4 | TS=clinical trial* OR TS=research design OR TS=comparative stud* OR TS=evaluation stud* OR TS=controlled trial* OR TS=follow-up stud* OR TS=prospective stud* OR TS=random* OR TS=placebo* OR TS=(single blind*) OR TS=(double blind*) | 3,352,507 |
| #5 | #1 AND #2 AND #3 AND #4 | 32 |
| **Chinese Biomedical Literature Database** | | |
| Number | Query | Results |
| #1 | "认知功能障碍"[加权:扩展] | 8,948 |
| #2 | ("MCI"[常用字段:智能] OR "轻度认知障碍"[常用字段:智能] OR "轻度认知损害"[常用字段:智能] OR "轻度认知损伤"[常用字段:智能] OR "轻度认知缺损"[常用字段:智能] OR "轻度认知减退"[常用字段:智能] OR "轻度认知受损"[常用字段:智能] OR "轻微认知损害"[常用字段:智能] OR "轻度神经认知障碍"[常用字段:智能] OR "轻度认知功能障碍"[常用字段:智能] OR "轻度认知功能损害"[常用字段:智能] OR "轻度认知功能损伤"[常用字段:智能] OR "轻度认知功能缺损"[常用字段:智能] OR "轻度认知功能减退"[常用字段:智能] OR "轻度认知功能受损"[常用字段:智能] OR "轻微认知功能损害"[常用字段:智能] OR "轻度神经认知功能障碍"[常用字段:智能] OR "认知障碍"[常用字段:智能] OR "认知损害"[常用字段:智能] OR "认知损伤"[常用字段:智能] OR "认知缺损"[常用字段:智能] OR "认知减退"[常用字段:智能] OR "认知受损"[常用字段:智能] OR "神经认知障碍"[常用字段:智能] OR "认知功能障碍"[常用字段:智能] OR "认知功能损害"[常用字段:智能] OR "认知功能损伤"[常用字段:智能] OR "认知功能缺损"[常用字段:智能] OR "认知功能减退"[常用字段:智能] OR "认知功能受损"[常用字段:智能] OR "神经认知功能障碍"[常用字段:智能]) | 92,426 |
| #3 | ("汤剂"[常用字段:智能] OR "煎剂"[常用字段:智能] OR "中西医"[常用字段:智能] OR "中医"[常用字段:智能] OR "中医药"[常用字段:智能] OR "中药"[常用字段:智能] OR "草药"[常用字段:智能] OR "中草药"[常用字段:智能] OR "中成药"[常用字段:智能] OR "成药"[常用字段:智能] OR "方剂"[常用字段:智能] OR "汤药"[常用字段:智能] OR "复方"[常用字段:智能] OR "自拟方"[常用字段:智能]) | 1,671,318 |
| #4 | ("多奈哌齐"[常用字段:智能] OR "盐酸多奈哌齐"[常用字段:智能] OR "安理申"[常用字段:智能] OR "多奈派齐"[常用字段:智能] OR "三水草酸多奈哌齐"[常用字段:智能]) | 3,671 |
| #5 | ("随机"[全部字段:智能] OR "盲法"[全部字段:智能] OR "安慰剂"[全部字段:智能]) | 1,884,449 |
| #6 | #1 AND #2 AND #3 AND #4 AND #5 | 679 |
| **Chinese Scientific Journal Database** | | |
| Number | Query | Results |
| #1 | M=(MCI OR 轻度认知障碍 OR 轻度认知损害 OR 轻度认知损伤 OR 轻度认知缺损 OR 轻度认知减退 OR 轻度认知受损 OR 轻微认知损害 OR 轻度神经认知障碍 OR 轻度认知功能障碍 OR 轻度认知功能损害 OR 轻度认知功能损伤 OR 轻度认知功能缺损 OR 轻度认知功能减退 OR 轻度认知功能受损 OR 轻微认知功能损害 OR 轻度神经认知功能障碍 OR 认知障碍 OR 认知损害 OR 认知损伤 OR 认知缺损 OR 认知减退 OR 认知受损 OR 神经认知障碍 OR认知功能障碍OR认知功能损害OR认知功能损伤OR认知功能缺损OR认知功能减退OR认知功能受损OR神经认知功能障碍) | 26,274 |
| #2 | R=(中药 OR 草药 OR 中草药 OR 中成药 OR 成药 OR 方剂 OR 汤药 OR 复方 OR 自拟方 OR汤剂 OR 煎剂 OR 中西医 OR 中医 OR 中医药) | 1,036,935 |
| #3 | R=(多奈哌齐 OR 盐酸多奈哌齐 OR 安理申 OR 多奈派齐 OR 三水草酸多奈哌齐) | 3,616 |
| #4 | U=(随机 OR 盲法 OR 安慰剂) | 132,328 |
| #5 | #1 AND #2 AND #3 AND #4 | 71 |
| **Chinese National Knowledge Infrastructure** | | |
| Number | Query | Results |
| #1 | SU=('MCI'+'轻度认知障碍'+'轻度认知损害'+'轻度认知损伤'+'轻度认知缺损'+'轻度认知减退'+'轻度认知受损'+'轻微认知损害'+'轻度神经认知障碍'+'轻度认知功能障碍'+'轻度认知功能损害'+'轻度认知功能损伤'+'轻度认知功能缺损'+'轻度认知功能减退'+'轻度认知功能受损'+'轻微认知功能损害'+'轻度神经认知功能障碍'+'认知障碍'+'认知损害'+'认知损伤'+'认知缺损'+'认知减退'+'认知受损'+'神经认知障碍'+'认知功能障碍'+'认知功能损害'+'认知功能损伤'+'认知功能缺损'+'认知功能减退'+'认知功能受损'+'神经认知功能障碍') | 33,762 |
| #2 | SU=('中药'+'草药'+'中草药'+'中成药'+'成药'+'方剂'+'汤药'+'复方'+'自拟方'+'汤剂'+'煎剂'+'中西医'+'中医'+'中医药') | 1,233,463 |
| #3 | SU=('多奈哌齐'+'盐酸多奈哌齐'+'安理申'+'多奈派齐'+'三水草酸多奈哌齐') | 3,221 |
| #4 | FT=('随机'+'安慰剂'+'盲法') | 9,454,270 |
| #5 | #1 AND #2 AND #3 AND #4 | 46 |
| **Wanfang Database** | | |
| Number | Query | Results |
| #1 | 主题:(MCI or 轻度认知障碍 or 轻度认知损害 or 轻度认知损伤 or 轻度认知缺损 or 轻度认知减退 or 轻度认知受损 or 轻微认知损害 or 轻度神经认知障碍 or 轻度认知功能障碍 or 轻度认知功能损害 or 轻度认知功能损伤 or 轻度认知功能缺损 or 轻度认知功能减退 or 轻度认知功能受损 or 轻微认知功能损害 or 轻度神经认知功能障碍 or 认知障碍 or 认知损害 or 认知损伤 or 认知缺损 or 认知减退 or 认知受损 or 神经认知障碍 or 认知功能障碍 or 认知功能损害 or 认知功能损伤 or 认知功能缺损 or 认知功能减退 or 认知功能受损 or 神经认知功能障碍) | 98,356 |
| #2 | 主题:(中药 or 草药 or 中草药 or 中成药 or 成药 or 方剂 or 汤药 or 复方 or 自拟方 or 汤剂 or 煎剂 or 中西医 or 中医 or 中医药) | 2,321,418 |
| #3 | 主题:(多奈哌齐 or 盐酸多奈哌齐 or 安理申 or 多奈派齐 or 三水草酸多奈哌齐) | 5,249 |
| #4 | 全部:(随机 or 盲法 or 安慰剂) | 3,375,218 |
| #5 | #1 AND #2 AND #3 AND #4 | 238 |
