# Supplementary figures and images for: Donepezil combined with traditional Chinese medicine has promising efficacy on mild cognitive impairment: a systematic review and meta-analysis
Source: Front Neurosci. 2023 Jul 5;17:1206491. doi: 10.3389/fnins.2023.1206491 (PMC10354366; doi:10.3389/fnins.2023.1206491)

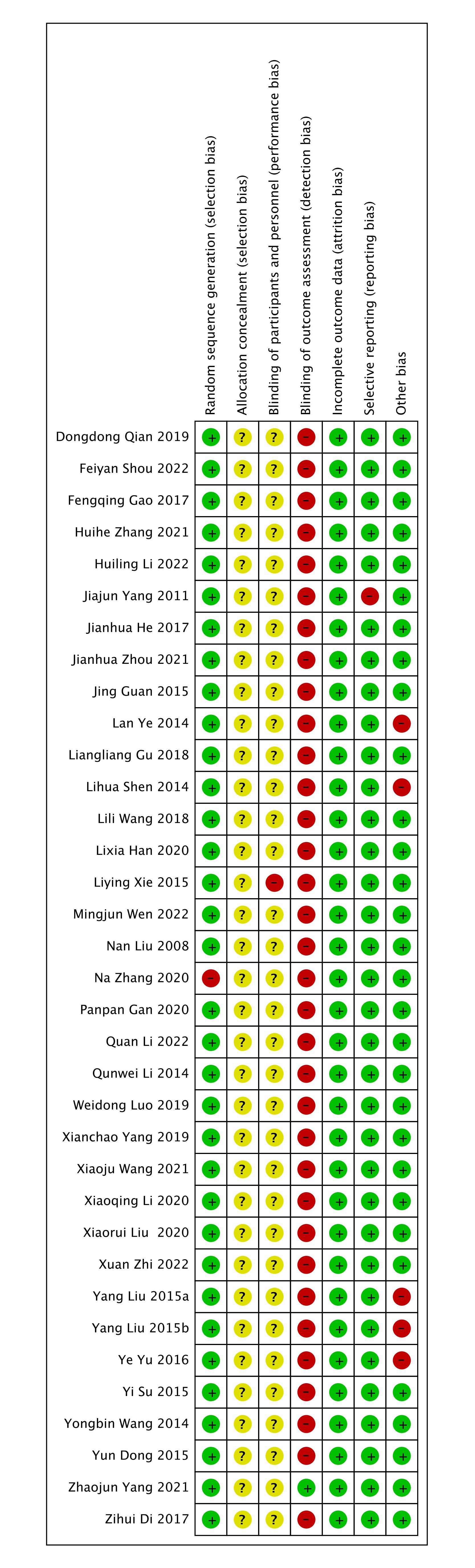

Supplement: Supplementary Figure 1 — Risk of bias for individual quality. [file Image_1.JPEG]

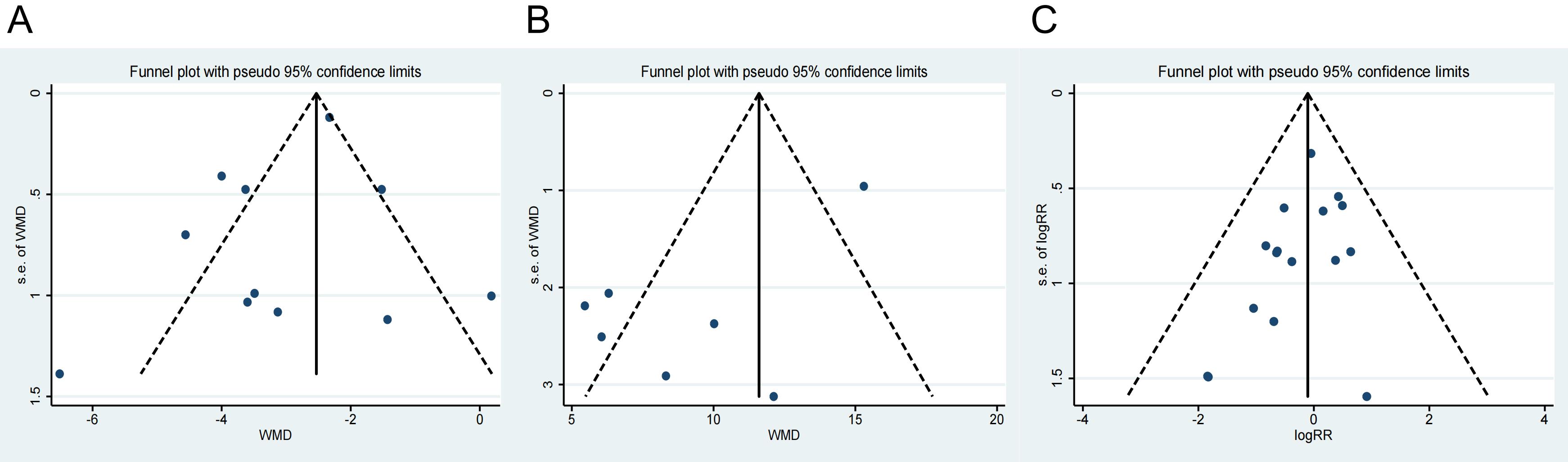

Supplement: Supplementary Figure 2 — Funnel plot of secondary outcomes. [file Image_2.JPEG]

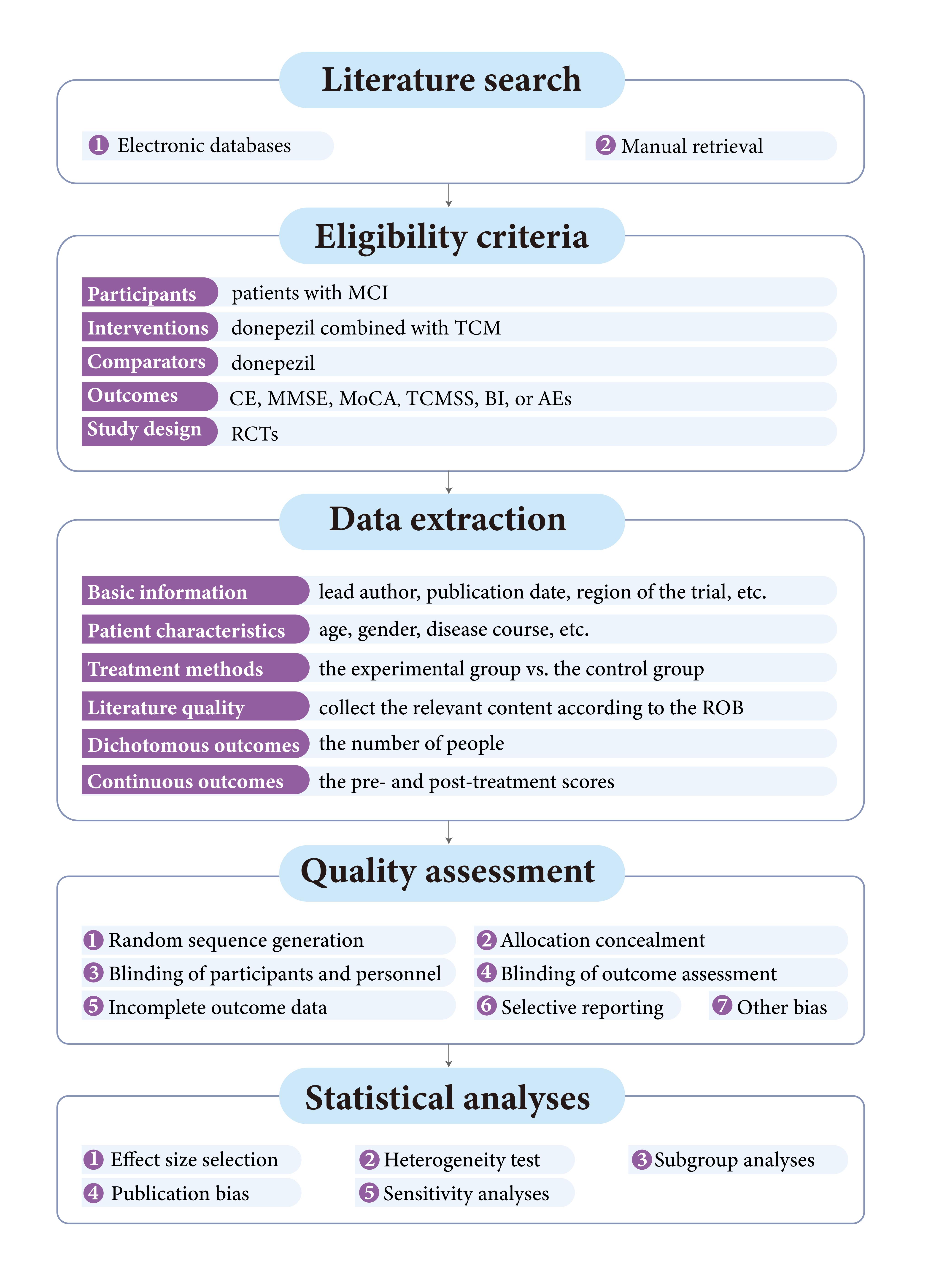

Supplement: Supplementary Figure 3 — Flowchart of method implementation. [file Image_3.JPEG]
